# Global Transcriptome Analysis Reveals Distinct Aluminum-Tolerance Pathways in the Al-Accumulating Species *Hydrangea macrophylla* and Marker Identification

**DOI:** 10.1371/journal.pone.0144927

**Published:** 2015-12-14

**Authors:** Haixia Chen, Changping Lu, Hui Jiang, Jinhui Peng

**Affiliations:** College of Horticulture and landscape, Hunan Agriculture University, Changsha, 410128, People republic of China; CSIR-National Botanical Research Institute, INDIA

## Abstract

Hydrangea (*Hydrangea macrophylla*) is a well known Al-accumulating plant, showing a high level of aluminum (Al) tolerance and accumulation. Although the physiological mechanisms for detoxification of Al and the roles of Al in blue hydrangea sepals have been reported, the molecular mechanisms of Al tolerance and accumulation are poorly understood in hydrangea. In this study, we conducted a genome-wide transcriptome analysis of Al-response genes in the roots and leaves of hydrangea by RNA sequencing (RNA-seq). The assembly of hydrangea transcriptome provides a rich source for gene identification and mining molecular markers, including single nucleotide polymorphism (SNP) and simple sequence repeat (SSR). A total of 401,215 transcripts with an average length of 810.77bp were assembled, generating 256,127 unigenes. After annotation, 4,287 genes in the roots and 730 genes in the leaves were up-regulated by Al exposure, while 236 genes in the roots and 719 genes in the leaves were down-regulated, respectively. Many transporters, including MATE and ABC families, were involved in the process of Al-citrate complex transporting from the roots in hydrangea. A plasma membrane Al uptake transporter, Nramp aluminum transporter was up-regulated in roots and leaves under Al stress, indicating it may play an important role in Al tolerance by reducing the level of toxic Al. Although the exact roles of these candidate genes remain to be examined, these results provide a platform for further functional analysis of the process of detoxification of Al in hydrangea.

## Introduction

Aluminum (Al) is the most abundant metal in the earth’s crust and is a toxic element for plants. All over the world, up to 40% ~50% of cultivable land is acidic and Al^3+^ toxicity is a major limiting factor for crop production [1]. In acidic soil, high concentrations of Al are existed and inhibit the root growth [2]. It also increases the levels of reactive oxygen species (ROS), which targets the plasma membrane and interacts with lipid components to initiate lipid peroxidation [3]. Therefore, enhancing Al tolerance of crops is a key to increase crop productivity on acidic soils. Elucidation of the detoxify strategies of some Al-accumulating plants will help us to increase the Al tolerance for crops. The Al-tolerance plant species can detoxify Al internally and externally [3, 4]. The root, and more specifically, the root tip, is the primary site of Al^3+^ toxicity, and the majority (~90%) of the Al^3+^ resides in the root cell wall [5]. To date, Al-activated secretion of organic acid from root apex is a well-documented mechanism of Al internal detoxification [6]. Different plants secrete different organic acids such as malate, citrate and oxalate to chelate Al and thereby attenuate Al^3+^ toxicity [7]. However, the process leading to the gene expression in the secretion and genes of Al response and tolerance remains not fully understood.

Hydrangea (*Hydrangea macrophylla*) is a well known Al-accumulating plant, and can become blue sepal color when it’s cultivated in acidic soil. Hydrangea plants can accumulate 5 mg Al g ^-1^ dry weight in the leaves within several months [8]. Aluminum, as Al^3+^, is not available to the hydrangea roots in basic or neutral soils, because it forms aluminum hydroxide and other insoluble precipitates. The main Al species in the cell sap of hydrangea leaves is the 1:1 complex of Al and citrate [9]. In acidic soils, Al^3+^ becomes available to roots, stimulating the hydrangea roots to exude citrates. The citrates form complexes with Al^3+^ and then the aluminum citrate complexes enter the roots and transport throughout the shrub [8].

Previous studies on aluminum in hydrangea were mainly about the sepals color changes [10, 11]. Few Al tolerance genes have been cloned and the corresponding molecular mechanism remains unclear in hydrangea, although the gene expression for Al tolerance and accumulation in several plants, including rice bean (*vigna umbellata*) [12], common buckwheat (*Fagopyrum esculentum*) [13] and tartary buckwheat (*Fagopyrum tataricum*) [7], were investigated. Therefore, identification of Al responsive genes associated with Al stress would provide valuable information for the detoxification mechanism of Al in hydrangea. However, as hydrangea is a non-model plant without the genome information, it will be a challenge to uncover the molecular mechanism of how the hydrangea receives the signal of Al stress and the expression of downstream genes, which finally lead to the physiological response.

Recently, RNA sequencing (RNA-Seq), a high-throughput sequencing method, is developed to analysis the transcriptome for both having or without genome information. It’s an efficient tool to promise simultaneous estimation of abundance and new transcript discovery [14]. RNA-Seq has been applied for transciptome analysis in many plant species, including *Youngia japonica* [15], sugarcane [16] and buckwheat [7, 13]. In this study, we used the RNA-Seq technique to analyze the transcriptome of roots and leaves of hydrangea exposed or not to Al, for identifying the differentially expressed genes. The aim is to identify Al responsive genes and new insight of molecular mechanisms of Al^3+^ toxicity and tolerance in hydrangea.

## Materials and Methods

### Plant materials

The *Hydrangea macrophylla* cultivars were cultivated in the garden of department of ornamental horticulture, Hunan Agricultural University. Cuttings of *Hydrangea macrophylla* cultivar “Lavbla” were subjected to hydroponic culture for growing. The solution contained KNO_3_ (1.0mM), Ca (NO_3_)_2_ (4mM), MgSO_4_ (1mM), KH_2_PO_4_ (1mM), NaFeEDTA (10μM), H_3_BO_3_ (50μM), MnSO_4_ (0.5μM), ZnSO_4_ (0.4μM), CuSO_4_ 0.5μM, (NH_4_)_6_Mo_7_O_24_ (1 μM). The solution was changed every day. The Al stress was treated as previous described [7, 13]. When the new roots grew, the cuttings were exposed to a 0.5mM CaCl_2_ solution (PH 4.5) containing 50 μM AlCl_3_. At the same time, the control cuttings groups grew in the same solution (0.5mM CaCl_2_, PH 4.5) only without AlCl_3_. After 4h, the roots (3cm from the root tip) and the leaves were harvested and frozen in liquid nitrogen and stored at -80°C for further use.

### RNA extraction, library construction and sequencing

Total RNA were extracted from the roots and leaves using an RNeasy Plant Mini Kit (QIAGEN) according to the manufacturer’s protocol. Messenger RNAs (mRNAs) from the total RNA were isolated using Oligo (dT) and were randomly cleaved into short fragments. Then the first strand cDNAs were synthesized with random hexamer primers and followed by second strand cDNAs synthesis using DNA polymerase I (New England BioLabs) and RNase H (Invitrogen). After end repair, adaptor ligation, and index codes adding for each sample, PCR amplification was performed. The quality and quantity of the libraries were detected using an Agilent 2100 Bioanalyzer and an ABI real time RT-PCR system. The qualified cDNA libraries were carried out for sequencing by an Illumina HiSeq 2500 platform with PE100. The raw sequence data obtained have been deposited at the NCBI in the Short Read Archive (SRA) database under the accession number: SRP061814.

### Sequence data analysis and De novo assembly

Raw reads were quality-checked with FastQC package, and adaptor sequences and low quality reads were removed. We carried out a stringent filtering criterion to minimize the effects of sequencing errors during the assembly. Firstly, bases with phred quality score lower than 20 and reads length short than 50bp would be discarded. Secondly, reads of 70% bases in a read having high phred quality scores (≥20) will be used for assembly. Thirdly, only the paired-end reads were used for further assembly. The obtained clean reads of all four samples were assembled by Trinity (Release 2013-07-08) using a paired-end model [17]. To annotate the assembled transcripts, BLASTx searches (E-value <1e-5) were performed against the protein databases, including NCBI non-redundant protein (NR) database, Swiss-Prot, Kyoto Encyclopedia of Genes and Genomes (KEGG) pathway database and Clusters of eukaryotic Orthologous Groups of proteins (KOG) database. The transcripts abundance was normalized by the reads per kilobase of transcript per million mapped reads (RPKM) value using the RSEM (RNASeq by Expectation Maximization) package [18]. And those transcripts with RPKM value equal or larger than 0.1 were defined as expressed. All the unigenes were translated into potential proteins according to ORF prediction by Getorf (http://emboss.sourceforge.net/apps/cvs/emboss/apps/getorf).

### Differential expression analysis and GO and KEGG enrichment analysis

The clean reads of each sample were mapped back to assembled contigs by bowtie2 [19]. The assembled contigs with more than 10 reads mapped were subjected to differential expression analysis. The expression difference of each transcript between different samples was calculated basing on the MARS (MA-plot-based method with Random Sampling) model using the DEGseq package. FDR (false discovery rate) value less than 0.01 and |log2(fold change)|≥2 were recognized as the significance of gene expression difference. Functional annotations of the differential unigenes were performed to search against the NR, Swiss-Prot, GO (Gene Ontology) and KOG database. The results of GO annotations were submitted to WEGO for GO classification. And GO functional enrichment and KEGG pathway enrichment analysis were also tested at a significance cutoff of p-value ≤0.01. To extract the STOP1/ART1-regulated gene homologs from hydrangea contigs, tBLASTx was performed. The aligned genes with the highest scores and the lowest E-value were selected as homolog genes in the list.

### Validation of RNA-seq by quantitative Real time RT-PCR

For the quantitative real time RT-PCR (qPCR) of the mRNAs, 1 μg of total RNA was used to synthesize the cDNA using the RevertAid First Strand cDNA Synthesis Kit (Fermentas). Quantitiative PCR was performed using the FastStart Universal SYBR Green Master (Roche) according to the manufacturer’s instruction on the StepOne plus Real time PCR Platform (Applied Biosystems). The qPCRs were carried out with the following protocol: 95°C for 10min, followed by 40 cycles of 95°C for 15s, and at 60°C for 60s. The 18S rRNA which is one of the reference genes in Hydrangea was used as the internal control [11]. After the amplification, the melting curve was determined for specific product. Three independent biological replicates for each sample and three technical replicates for each biological replicate were analyzed. All the primers used are listed in S1 Table. Significant differences of the expression level between roots (-Al) and roots (+Al) or leaves (-Al) and leaves (+Al) were evaluated using Student’s *t* test.

### Mining of SNP and Simple Sequence Repeat (SSR)

SNPs of the transcripts between different samples were detected using SOAPsnp [20]. The filter criteria were as follow: (1) the total coverage and the number of reads to cover a candidate SNP (>8 reads); (2) low phred quality (<25) reads from the coverage were removed; (3) frequency of mutated bases among all reads covering the position was higher than 30%.

SSR markers were identified by the MISA (MIcroSAtellite identification tool) Perl script (http://pgrc.ipk-gatersleben.de/misa). The settings for minimum number of repeats were mono-, di-, tri-, tetra-, penta-, and hexa-nucleotide motifs with numbers of uninterrupted repeat units more than 10, 6, 5, 5, 5, and 5, respectively. For compound SSRs, the maximum distance between the two SSR was 50bp.

## Results

### De novo assembly of the transcripts and annotation

**Fig 1:**
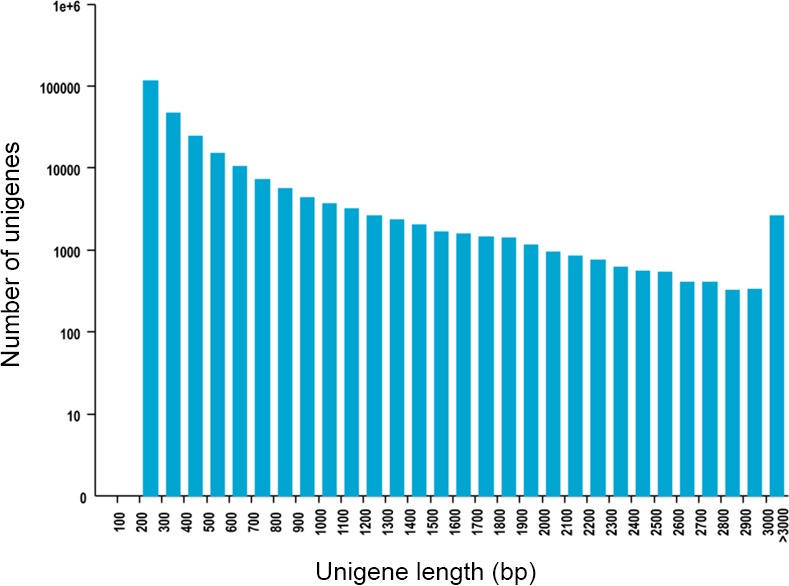
Distribution of the length of transcript assembly unigenes.

The total reads of hydrangea roots and leaves were ranging from 17~21 million, depending on the tissues and treatments (Table 1). Using the de novo assembly program Trinity, the consensus sequences were constructed. As a result, a total of 8,919,586 contigs from the four samples were assembled (Table 2). Of them, a total of 401,215 transcripts and 256,127 unigenes were found. The assembled unigenes had a length distribution from 201 to 15,907 with an average length of 531.98bp (Fig 1 and Table 2). Recently, transcriptome sequencing of buckwheat (*F*. *tataricum*) assembled 148,734 contigs and 84,516 unigenes [13]. Compared with their results, we produced more contigs (8,919,586) and more unigenes (256,127). Therefore, the assembled contigs in this study can provide a useful resource for further research of hydrangea.

**Table 1:** Summary of short-read data from hydrangea produced by Illumina sequencing.

Sample	Total reads	Total mapping reads	Unique mapping reads
Root (-Al)	20,249,505	15,293,610(75.53%)	8,388,933(54.85%)
Root (+Al)	17,307,862	12,037,300(69.55%)	6,854,798(56.95%)
Leaf (-Al)	21,278,791	17,578,600(82.61%)	9,749,241(55.46%)
Leaf (+Al)	18,670,322	15,190,262(81.36%)	8,540,894(56.23%)

**Table 2:** The length distribution of assembled contigs, transcripts and unigenes.

Length range	Contigs	Transcripts	Unigenes
200–300	8,755,051(98.16%)	130,237(32.46%)	113,460(44.30%)
300–500	83,954(0.94%)	92,631(23.09%)	71,393(27.87%)
500–1000	49,990(0.56%)	76,458(19.06%)	42,217(16.48%)
1000–2000	22,772(0.26%)	64,340(16.04%)	20,838(21.77%)
>2000	7,819(0.09%)	37,549(9.36%)	8,219(3.21%)
Total number	8,919,586	401,215	256,127
Total length	581,831,307	325,293,471	136,253,549
N50 length	79	1,436	683
Mean length	65.23	810.77	531.98

For annotation, the 256,127 unigenes were subjected to BLASTx searches against the sequences in the NR, Swiss-Prot and KOG databases (E-value ≤ 1e-5). As a result, a total of 74,167 sequences were annotated, of them, 73,853(77.16%), 57,278 (59.84%) and 28,596 (29.88%) unigenes were aligned against the three protein databases, respectively. GO and KEGG analysis were also performed, having 47,670 and 18,270 unigenes, respectively (Table 3).

**Table 3:** Annotation of Unigenes were searched against from NR, SwissProt, GO, KOG, KEGG by BLAST.

Annotated databases	All sequence	> = 300bp	> = 1000bp
KOG	28,596	17,029	11,567
GO	47,670	28,697	18,973
KEGG	18,270	11,429	6,841
Swiss-Prot	57,278	34,059	23,219
NR	73,853	47,143	26,710
All	74,167	47,410	26,757

### Global effect of Al stress on gene expression

**Fig 2:**
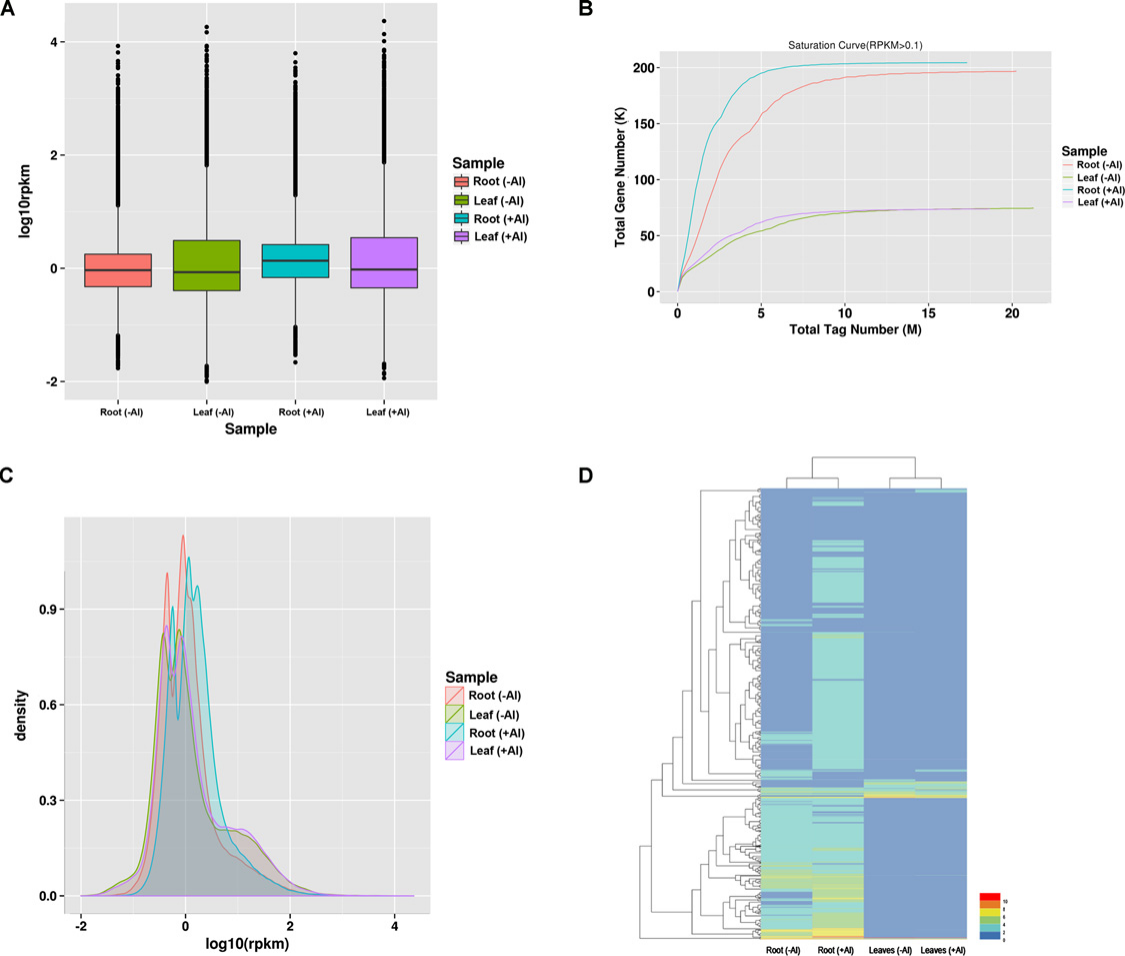
The overview of differential expression between no Al and Al stress in roots and leaves in hydrangea. (A) Boxplots of the DEGs in the four samples; (B) Sequencing saturation level analysis of DEGs in the four samples; (C) Density distribution of the differentially expressed genes (DEGs) in the four samples; (D) heat map of the DEGs in roots and leaves between Al(-) and Al (+).

To get insight into the gene expression patterns in roots and leaves of hydrangea under Al stress, RPKMs were calculated for each sample and all the unigenes were annotated (Fig 2, S2 Table and S3 Table). The results showed that the roots had more differentially expressed genes (DEGs) than that in leaves (Fig 2A, 2B and 2C) and many genes were up regulated in roots or leaves under Al stress (Fig 2D). The unigene abundance was quantified using Bowtie and RSEM package. The significant DEGs were judged by |log2 (fold change)| ≥2 and FDR value less than 0.01. In the roots, compared with control (-Al), 4,287 and 236 annotated genes were up-regulated and down-regulated in roots (+Al), respectively (S4 Table). In the leaves, the number of genes up- and down- regulated by Al was 730 and 719, respectively (S4 Table). Although the down- regulated genes in roots and DEGs in leaves were lower than buckwheat [13], the results in this study were similar with previous reports, having more up-regulated genes in roots under Al stress.

### Functional classification of the Al stress genes

**Fig 3:**
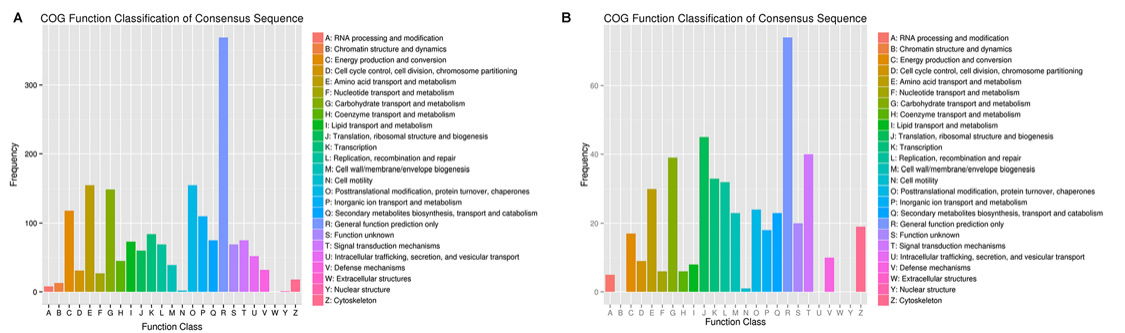
Histogram of KOG classification of differential expressed unigenes in roots (A) and leaves (B) under Al stress in hydrangea.

To identify the genes that are differentially expressed under Al stress, a functional categorization was carried out by GO and KEGG functional annotation, respectively (Table 3). The annotated unigenes were also compared with the eukaryotic orthologous groups (KOG) database for functional prediction and classification. In total, 28,596 annotated putative proteins were classified into 25 KOG groups (Fig 3). Among the 25 categories, “Extracellular structures” was not represented in both hydrangea roots and leaves under Al stress. In roots, the larger groups included “Posttranslational modification, protein turnover, chaperones”, “Amino acid transport and metabolism” and “Carbohydrate transport and metabolism”. Unlike the roots, three groups “Translation, ribosomal structure and biogenesis”, “Signal transduction mechanisms” and “Carbohydrate transport and metabolism” were represented as the major number groups in leaves of hydrangea.

**Fig 4:**
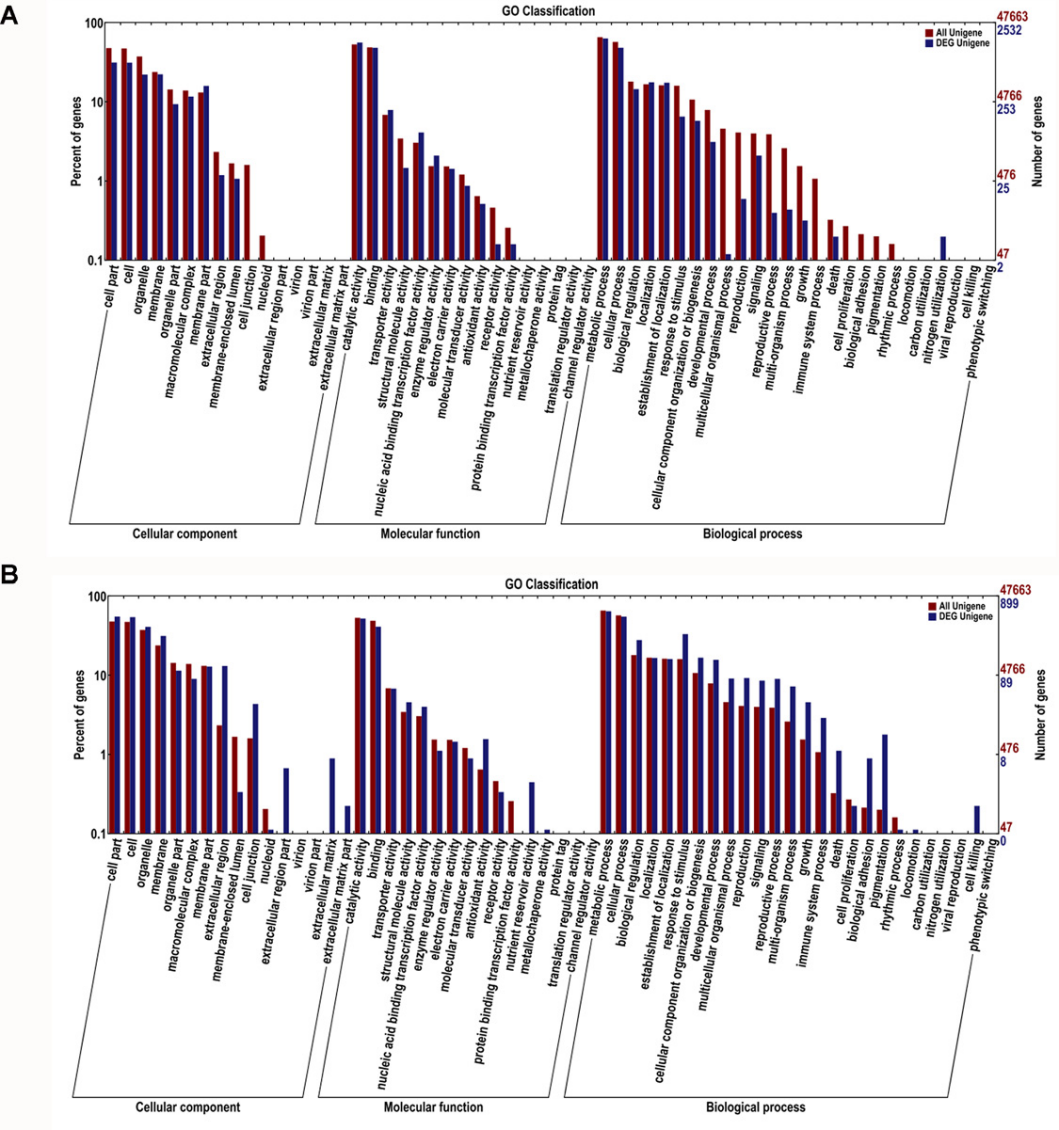
Gene Ontology classifications of differential expressed unigenes in roots (A) and leaves (B) under Al stress in hydrangea.

GO analysis showed that the total of 47,670 predicted proteins were categorized into 57 functional groups under three main divisions (Cellular Components, Molecular Functions and Biological Processes) (Fig 4). Among DEGs by Al in the roots, the “membrane part” in cellular component and “transporter activity” or “enzyme regulator activity” in molecular functions was overrepresented. Particularly, “nitrogen utilization” in biological processes was only represented in Al stress roots (S5 Table and Fig 4A). In the leaves, among the Al-induced up- and down-regulated genes, “extracellular matrix”, “extracellular region” and “membrane” in cellular component were the predominant groups. In the molecular functions category, “nutrient reservoir activity” and “antioxidant activity” were the most representative ones. With regard to biological processes, the predominant categories were “cell killing”, followed by “death”, “immune system process” and “response to stimulus” (S6 Table and Fig 4B).

**Fig 5:**
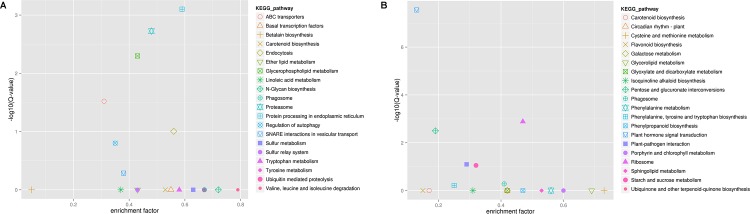
KEGG enrichment analysis of differential expressed unigenes in roots (A) and leaves (B) under Al stress in hydrangea.

To determine whether the genes involved in specific metabolic or signal transduction pathways, the DEGs were search against the KEGG pathway database. By comparing the control without Al stress, all DEGs were assigned to 107 and 79 pathways in roots and leaves under Al stress, respectively (S7 Table). The top 20 obviously enriched pathways were shown in Fig 5. These DEGs were enriched in “ABC transporters”, “signal transduction”, and “lipid metabolism”, including “Ether lipid metabolism”, “Glycerophospholipid metabolism”, “Sphingolipid metabolism” (Fig 5). The significant pathways in roots were “Protein processing in endoplasmic reticulum” (gene number [N] = 72), followed by “Proteasome” (N = 37) and “glycerophospholipid metabolism” (N = 26) (Fig 5A and S7 Table). In Al stress leaves, more DEGs were enriched in the pathways “plant hormone signal transduction” (N = 16), “ribosome” (N = 34) and “pentose and glucuronate interconversions” (N = 9) (Fig 5B and S8 Table).

### Validation of RNA-seq data by quantitative real-time RT-PCR

**Fig 6:**
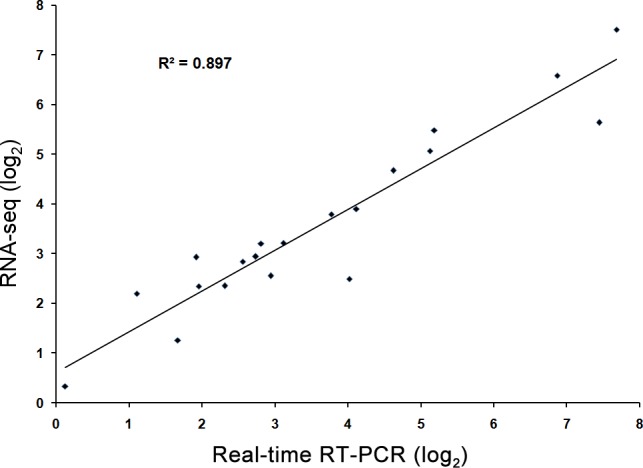
Validation of the expression data from RNA-seq analysis via quantitative real-time RT-PCR (qPCR) analysis. Twenty genes exhibiting diverse expression profiles in the RNA-seq data were chosen for qPCR analysis. Average value of each RNA-seq expression data was plotted against that from qPCR and fit into a linear regression. Both x- and y-axes were shown in log_2_ scale.

The expression of each gene from the RNA-seq data was calculated by RPKM. To verify the RNA-seq expression data, we selected 20 genes displaying diverse expression profiles in the roots and leaves for quantitative real-time RT-PCR analysis. A good correlation (R^2^ = 0.89) was observed between RNA-seq data and qPCR data (Fig 6). These results confirmed the high reliability of the RNA-seq data obtained in the present study.

### Expression profile of STOP/ART1-regulated genes and organic acid metabolism

STOP1 (Sensitive to Proton Rhizotoxicity1), a C2H2 zinc finger-type transcription factor for Al response gene expression, was firstly identified in Arabidopsis [21]. And the AtSTOP1 homolog in rice (*Oryza sativa*) is ART1 (Al Resistance Transcription Factor1) [22]. It has been reported that STOP1/ART1 regulates the expression of three major Al tolerance genes: Aluminum activated Malate Transporter1 (ALMT1), ALUMINUMSENSITIVE3 (AtALS3) and MATE (Multidrug and Toxic Compound Extrusion). In Arabidopsis, 43 genes are regulated by STOP1 [23, 24]. Among these regulated genes, 7 homologous genes were up-regulated in hydrangea roots (Table 4). Genes homologous to ATROP4 (rho-like GTP-binding protein 4) (c220714.graph_c0), PLT3 (probable polyol transporter 3) (c211750.graph_c1, c204065.graph_c0, c203825.graph_c0) and ATNADP-ME2 (malic enzyme 2) (c175032.graph_c0) were also up-regulated by Al (Table 4). However, their roles in Al tolerance were unknown.

**Table 4:** Comparison of expression profiling between STOP/ART-regulated genes in rice or Arabidopsis and corresponding genes in hydrangea roots.

RAP or TAIR ID^a^	Description^b^	Gene ID^c^	Fold change^d^
Os01g0860500	Chitinase	c219192.graph_c0	3.6
		c214980.graph_c0	3.4
		c149908.graph_c2	3.9***
Os04g0583500	Expansin-A10	c172142.graph_c0	-3.8
Os09g0479900	Subtilisin like serine protease	c214930.graph_c0	4.1
		c161922.graph_c0	4.2***
		c180217.graph_c0	-3.7
Os02g0755900	UDP-glucuronosyl/UDP-glucosyltranferase	c223402.graph_c0	3.3
Os10g0206800	OsFRDL2	c209989.graph_c0	4.0
		c166030.graph_c4	2.9
		c120666.graph_c0	3.2
Os02g0186800	Cytochrome P450 family protein	c92469.graph_c0	3.6
		c210342.graph_c3	4.3
		c193066.graph_c0	4.7
At2g45220	Similar to pectin esterase family protein	c155695.graph_c0	2.9
		c183224.graph_c0	3.4
At1g75840	ATROP4 (rho-like GTP-binding protein 4)	c220714.graph_c0	4.1
At2g23150	ATNRAMP3(manganese ion transporter)	c140176.graph_c0	3.9
At2g18480	PLT3 (probable polyol transporter 3)	c211750.graph_c1	5.9
		c204065.graph_c0	6.6
		c203825.graph_c0	6.2
At3g05400	SUGTL5 (sugar transporter ERD6-like 12)	c207258.graph_c2	3.5
		c155119.graph_c1	3.3
At3g12750	ZIP (zinc transporter 1)	c183609.graph_c0	4.2
		c217013.graph_c0	3.8
		c225267.graph_c0	3.7
At5g11670	ATNADP-ME2 (malic enzyme 2)	c175032.graph_c0	2.8

a, TAIR (The Arabidopsis Information Resourse) ID.

b, Description based on the TAIR-DB.

c, Hydrangea assembly unigenes ID.

d, Fold change of expression ratio of +Al treatment and–Al treatment with log2 normalization.

*** The expression level was validated by qPCR. The asterisks indicate significant differences between roots (+Al) and roots (-Al), as determined by Student’s *t* test (***P < 0.001).

**Fig 7:**
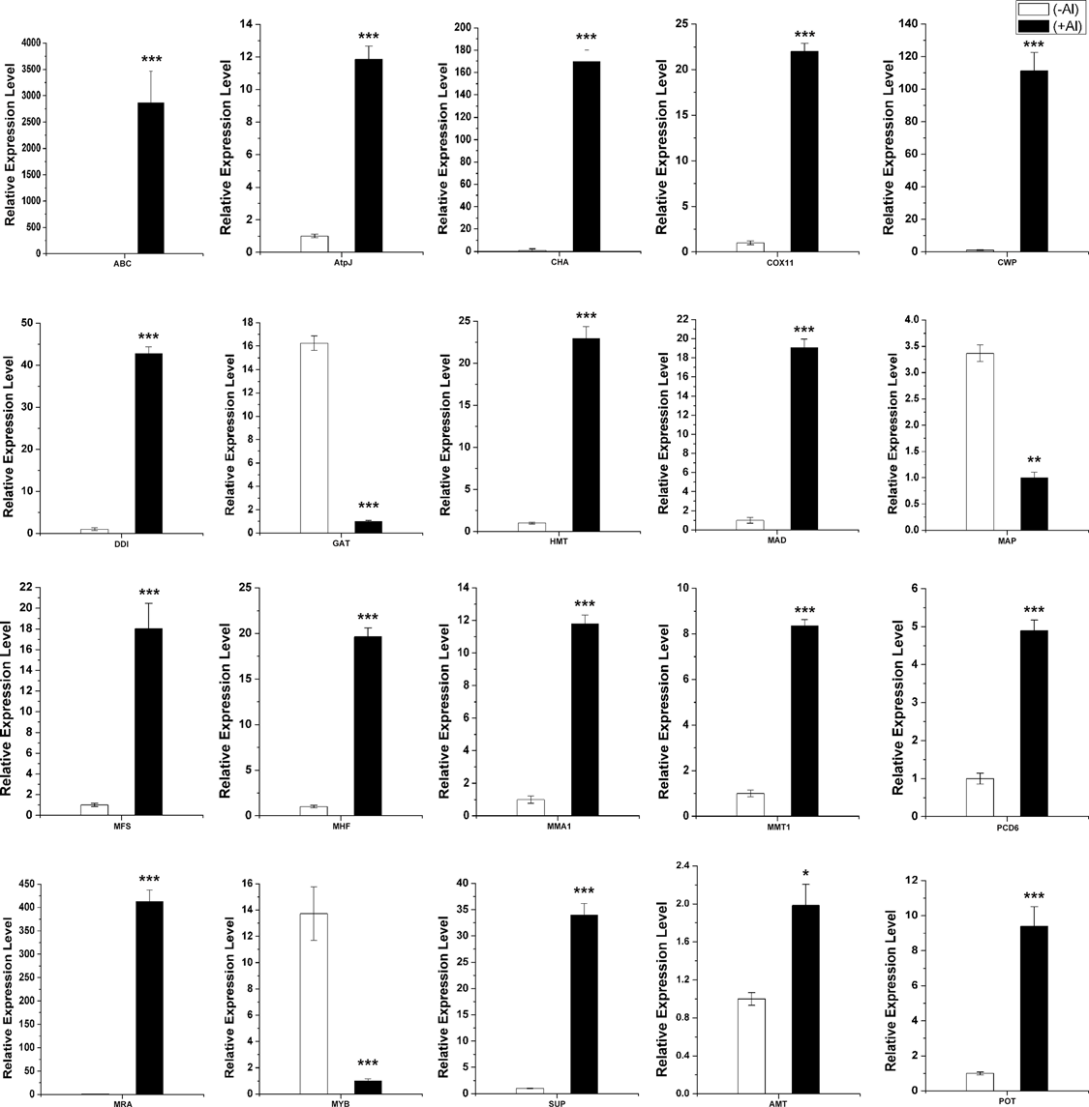
Expression analysis of differential expression unigenes in root and leaves under Al stress in hydrangea by quantitative real-time RT-PCR (qPCR). Eighteen genes in roots: ABC (c166032.graph_c0), ABC transporter B family member 12; AtpJ (c176296.graph_c0), ATP synthase subunit J; CHA (c149908.graph_c2), Chitinase-like protein; COX11 (c220486.graph_c0), Cytochrome c oxidase assembly protein COX11; CWP(c193058.graph_c1), Cell wall protein DAN4; DDI (c201184.graph_c0), DNA damage-inducible protein 1; GAT (c211725.graph_c0), Glucose/galactose transporter; HMT (c205800.graph_ c0),Heavy metal tolerance protein; MAD (c86593.graph_c0), Malate dehydrogenase; MAP(c119898.graph_c0), malate carrier protein; MFS(c219821. graph_c0), Major facilitator superfamily; MHF(c185758. graph_c0), Metal homeostasis factor ATX1; MMA1 (c152377.graph_c0), Mitochondrial metalloendopeptidase; MMT1(c142806.Graph_c0), Mitochondrial metal transporter 1; PCD6 (c127753.Graph _c0), Programmedcell death protein 6; MRA (c211816.graph_c0), Multidrug resistance- associated protein 1; MYB (c156038. graph_c0), Myb-like protein; SUP(c161922.graph_c0), Subtilisin-like proteinase Spm1. Two genes in leaves: AMT (c208330. graph_ c1), Aluminum-activated malate transporter; POT (c86493.graph_c0), Potassium transporter 1. The asterisks indicate significant differences between root (+Al) and root (-Al), or leaves (+Al) and leaves (-Al), as determined by Student’s *t* test (*P<0.05, **P < 0.01, ***P < 0.001).

Previous reports showed that ART1 regulated 32 genes in rice roots [22, 25]. Five homologous genes in hydrangea roots were also up-regulated by Al (fold change >2) (Table 4). Especially, three MATE genes (c209989.graph_c0, c166030.graph_c4, c120666.graph_c0) homologous to OsFRDL2 (FERRIC REDUCTASE DEFECTIVE LIKE2), were up-regulated more than 3-fold by Al (Table 4). The MATE genes from the citrate transporter AtFRD3 have been shown to be involved in transporting citrate [26]. Furthermore, MDH (malate dehydrogenase) (c86593.graph_c0), CS (citrate synthase) (c171733.graph_c0, c182066.graph_c1), key enzymes in the tricarboxylic acid (TCA) cycle, were up-regulated in roots (Fig 7). In the glycolate/glyoxylate pathway, genes encoding isocitrate lyase (c171402.graph_c1) and serine hydroxymethyltransferase (c191330.graph_c0) were up-regulated in roots. However, in the leaves, gene encoding malate dehydrogenase (c205150.graph_c2) was down-regulated. All the other genes involved in TCA cycle were not induced by Al in the present study. These results were consisted with other Al-accumulating plant, such as buckwheat [13]. In addition, we found three genes belonging to the MATE family were induced in roots and the OsFRDL4 homologous gene (c202686.graph_c0) was also up-regulated in leaves. Although Al-activated citrate secretion is not the Al-tolerance mechanism, citrate might be transported into the xylem for Al translocation [7, 27].

### Al-up-regulated transporter genes in roots and leaves

Transporters are required for Al-induced secretion of citrate and for Al uptake, sequestration to vacuoles [28]. In this study, 57 and 37 transporter genes were Al-induced in roots and leaves, respectively (Tables 5 and 6 and S9 Table). Many of these genes in roots (6) and leaves (6) belong to the ABC (ATP-binging cassette) transporter family (Table 5). Some other transporter genes induced by Al belonged to different types of transporter families (Tables 5 and 6 and S9 Table). For example, three genes similar to MFS (Major Facilitator superfamily protein) family have been reported to be involved in vacuolar sequestration of Al [13]. Genes showing high similarity to nitatre transporter, magnesium transporter, potassium transporter and sulfate transporter were also up-regulated by Al (Tables 5 and 6). In addition, vacuolar amino acid transporter genes, multispecific organic anion and peptide transporter genes were also up-regulated (Tables 5 and 6). Similar results were reported in buckwheat, implied that they might play important roles in Al tolerance. Although these transporters have not been reported to be associated with Al tolerance, investigation of them in the next step will help for further understanding the mechanism of Al tolerance in hydrangea.

**Table 5:** Al-induced transporter genes in hydrangea roots.

Gene ID^a^	Description^b^	Log2FC^c^	Category^d^
c203825.graph_c0	Polyol transporter 2	6.2	
c176926.graph_c0	Sphingoid long-chain base transporter	6.2	
c140176.graph_c0	Manganese transporter SMF1	3.9	
c166854.graph_c0	High-affinity glucose transporter	5.6	
c221976.graph_c0	Oligopeptide transporter 2	Only+Al	
c211750.graph_c1	Polyol transporter 5	5.9	
c217448.graph_c0	multispecific organic anion transporter 1	Only+Al	
c219821.graph_c0	Major facilitator superfamily protein	4.2	MFS famliy
c148474.graph_c0	H(+)/Cl(-) exchange transporter 3	3.8	
c217013.graph_c0	zinc transporter	3.8	
c148512.graph_c0	Vacuolar amino acid transporter 3	3.5	
c193777.graph_c0	Peptide transporter PTR2	3.8	
c187318.graph_c0	Uncharacterized MFS-type transporter	3.1	MFS famliy
c214350.graph_c0	Vacuolar calcium ion transporter	3.0	
c190249.graph_c1	Carboxylic acid transporter	4.0	
c225267.graph_c0	Zinc-regulated transporter 1	Only+Al	
c90732.graph_c0	Uncharacterized MFS-type transporter	4.5	MFS famliy
c155119.graph_c1	Sugar transporter STL1	3.2	
c200548.graph_c1	ATP-binding cassette transporter	3.4	ABC family
c151064.graph_c0	High-affinity nicotinic acid transporter	3.3	
c142806.graph_c0	Mitochondrial metal transporter 1	3.0***	
c130251.graph_c0	Fatty acid transporter	3.5	
c217697.graph_c0	Uncharacterized MFS-type transporter	2.9	MFS famliy
c148684.graph_c0	Magnesium transporter NIPA2	3.3	
c143855.graph_c0	Vacuolar amino acid transporter 2	3.1	
c170606.graph_c0	Multispecific organic anion transporter 1	Only+Al	
c230493.graph_c0	Multispecific organic anion transporter 2	3.5	
c90673.graph_c1	Iron-sulfur clusters transporter	Only+Al	
c183645.graph_c0	ABC1 family protein C15C4	3.2	ABC family
c101365.graph_c0	ATP-binding cassette sub-family A	Only+Al	ABC family
c145066.graph_c1	ATP-binding cassette sub-family D	3.9	ABC family
c166032.graph_c0	ABC transporter B family member 12	Only+Al***	ABC family
c192035.graph_c2	ATP-binding cassette transporter	4.1	ABC family
c198291.graph_c0	Nramp aluminum transporter 1	1.7	NRAMP family

a, Hydrangea assembly unigenes ID.

b, Description based on the BLASTx with NR and Swiss-Prot database.

c, Fold change of expression ratio of +Al treatment and–Al treatment with log2 normalization.

d, Transporter category.

*** The expression level was validated by qPCR. The asterisks indicate significant differences between roots (+Al) and roots (-Al), as determined by Student’s *t* test (***P < 0.001).

**Table 6:** Al-induced transporter genes in Hydrangea leaves.

Gene ID^a^	Description^b^	Log2FC^c^	Category^d^
c86493.graph_c0	Potassium transporter 1	2.9***	
c211481.graph_c0	ABC transporter	2.2	ABC family
c205928.graph_c0	Potassium transporter 2	1.2	
c208330.graph_c1	Aluminum-activated malate transporter	1.1	
c199526.graph_c0	Plastidic glucose transporter 2	1.4	
c207435.graph_c0	Sulfate transporter	3.2	
c181864.graph_c2	ABC transporter	1.6	ABC family
c186746.graph_c0	Sulfate transporter	1.8	
c205623.graph_c1	Nitrate transporter	1.0	
c198291.graph_c0	Nramp aluminum transporter 1	0.8	NRAMP family
c190578.graph_c0	Peptide transporter	1.2	
c156211.graph_c0	Equilibrative nucleotide transporter	5.3	
c204623.graph_c0	Sugar transporter	1.2	
c208984.graph_c0	MATE efflux protein	0.3	MATE family
c207435.graph_c1	Low affinity sulfate transporter 3	2.8	
c122554.graph_c0	Vacuolar iron transporter	2.4	
c202686.graph_c0	Citrate efflux MATE transporter	1.3	MATE family
c191802.graph_c0	Auxin transporter-like protein	1.3	
c179282.graph_c0	ABC transporter	1.8	ABC family
c204800.graph_c2	Amino acid transporter 6	1.3	
c187439.graph_c0	ABC transporter	2.0	ABC family
c205514.graph_c0	Nitrate excretion transporter	1.3	
c176091.graph_c0	Phosphate transporter	4.3	

a, Hydrangea assembly unigenes ID.

b, Description based on the BLASTx with NR and Swiss-Prot database.

c, Fold change of expression ratio of +Al treatment and–Al treatment with log2 normalization.

d, Transporter category.

*** The expression level was validated by qPCR. The asterisks indicate significant differences between leaves (+Al) and leaves (-Al), as determined by Student’s *t* test (***P < 0.001).

In rice, uptake of Al^3+^ is mediated by NRAT1 (NRAMP ALUMINUM TRANSPORTER1), a member of the NRAMP family. In the study, we also found a homolog gene of NRAT1 in our RNA-seq data of the roots and leaves, indicating its role in Al uptake (Table 5). Some members of MATE family can transport citrate to participate in Al tolerance. A gene similar to citrate efflux MATE transporter was also found to be up-regulated in leaves in this study (Table 6). Two up-regulated transporter genes mitochondrial metal transporter 1 (c142806.graph_c0), ABC transporter (c166032.graph_c0) in roots, Aluminum-activated malate transporter (c208330.graph_c1), and potassium transporter 1 (c86493.graph_c0) in leaves were further verified by qPCR analysis (Fig 7).

### Genes related to stress/defence, energy and cell wall organization in roots

A number of genes related to stress and energy metabolism were identified to be affected by Al (S10 Table). For example, stress response protein nst1 (c141215.graph_c1, c87075.graph_c0 and c173987.graph_c1), ATP synthase subunit 5 (c195438.graph_c0), AtpJ (c176296.graph_c0) and Atpα (c172514.graph_c0) were up-regulated in roots. Many genes related to oxidative stress, such as Cu/Zn superoxide dismutase (c145636.graph_c0, c199996.graph_c1) and L-ascorbate oxidase (c179819.graph_c0) were induced by Al. In the study, genes involved in cell wall organization were identified, in which three were cell wall integrity and stress response genes (c166600.graph_c0, c209786.graph_c0, c92982.graph_c0). Many cell wall proteins and vegetative cell wall proteins were also up-regulated in roots under Al stress (S10 Table). Some of these genes including cell wall protein DAN4 (c193058.graph_c1), cox11 (c220486.graph_c0) and AtpJ (c176296.graph_c0) were selected for qPCR analysis and confirmed their high expression level in roots (+Al) (Fig 7).

Genes related to other biological processes, including signal transduction, transcription, DNA damage and metal homeostasis, were induced by Al in hydrangea roots. For instance, many mitogen-activated protein kinases and Zinc finger transcription factors were up-regulated in roots (S10 Table). Moreover, DNA damage-inducible protein (c201184.graph_c0), metal homeostasis factor (c185758.graph_c0), metallothionein (c129315.graph_c0) and heavy metal tolerance protein (c171214.graph_c0) were also showed high expression in Al stress roots (S8 Table). Further qRT-PCR analysis verified these results (Fig 7).

### SSR and SNP mining

The assembled transcripts provide a rich source for potential genic-SSR markers, which are potentially linked with functional genes. Using MISA Perl script, a total of 15,899 SSRs with 1–6 repeat motifs and 1,318 compound SSR motifs were identified in 29,057 sequences (Table 7 and S11 Table). The largest proportion of SSRs consisted of di-nucleotide repeats (46.13%), followed by mono-nucleotide repeats (38.51%) (Table 7). The most abundant di-nucleotide and tri-nucleotide motifs were AG/CT (69.29% in di-SSRs) and AAG/CTT (26.62% in tri-SSRs), respectively (S11 Table).

**Table 7:** Classification of SSR repeats in the hydrangea transcriptome assembly.

**SSR data**	
Number of seqs searched	29,057
Total size of sequences (bp)	52,904,098
Number of SSRs	15,899
Number of SSR contining seqs	10,396
Number of seqs containing more than one SSR	3,792
Number of compound SSRs	1,318
**Distribution of SSR repeat types**	
Mono-nucleotides (≥10 repeats)	6123(38.51%)
Di-nucleotides (≥6 repeats)	7335(46.13%)
Tri-nucleotides (≥5 repeats)	2258(14.20%)
Tetra-nucleotides (≥5 repeats)	135(0.85%)
Penta-nucleotides (≥5 repeats)	23(0.14%)
Hexa-nucleotides (≥5 repeats)	25(0.16%)

**Fig 8:**
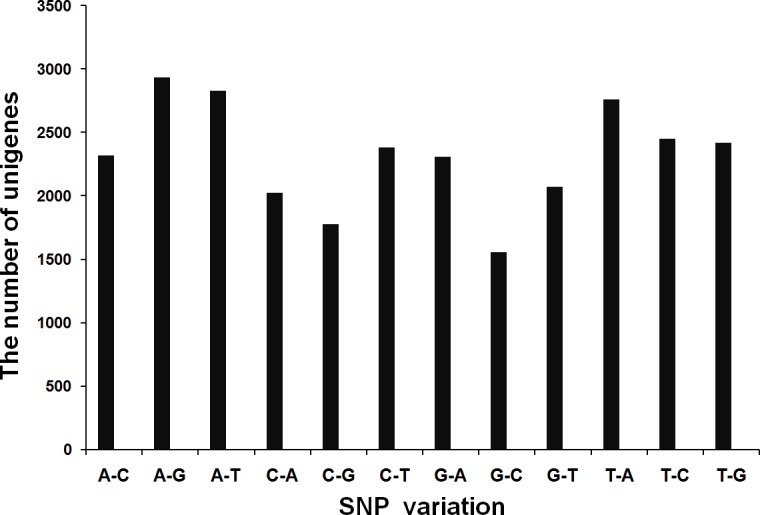
SNP distribution of different variation types in hydrangea.

Genic-SNPs are useful for linking to candidate genes or QTLs. In this study, 27,816 SNPs were identified in the four samples. The most abundance of base substitution was A/G (2931, 10.54%), followed by A/T (2826, 10.16%) (Fig 8). Although a stringent criteria were used to identify the SNPs (see Methods), the frequencies of SNPs in unigenes were detected highly (S12 Table).

## Discussion

Using high-throughput RNA sequencing in non-model plant hydrangea, a large amount of sequence data was generated. After *de novo* assembly of the sequences, nearly 130,237 transcripts were constructed in hydrangea (Table 1). Since the EST data of hydrangea available in NCBI is few, these results provided a large number of resources for gene discovery, quantification of transcripts in Al stress and the identification of putative SNPs and SSRs.

The differential expression analysis of RNA-seq data in control and Al stress revealed that more genes up-regulated in roots than leaves (S7 Table and S8 Table), which suggested that root might be more sensitive to Al stress at the primary time. And further investigation needed to validate the results. GO enrichment analysis showed that the DEGs in roots or leaves were significantly overrepresented in “Response to stimulus”, “Antioxidant activity”, “membrane”,”Extracellular region” and “death”. The results suggested that defensive genes and genes encoding extracellular-localized proteins, such as cell wall component, were preferentially induced in expression by Al stress. For KEGG pathway analysis, “Lipid metabolism” was significantly enriched in root and leaves. The enrichment of genes in the lipid metabolism pathway supported the observation that Al can interfere with the function of the plasma membrane. By contrast, genes in “ABC transporters”, “Proteasome” and “Protein processing in endoplasmic reticulum” pathways were only significantly enriched in roots, while genes in “Plant hormone signal transduction” were overrepresented in leaves but not in roots under Al stress. The DEGs were also subjected to KOG classification, showing different categories in roots and leaves except for “Carbohydrate transport and metabolism”. Therefore, the roots and leaves may possess different mechanism of Al responsiveness in hydrangea.

Recently, STOP1 or ART1 gene homologs from Eucalyptus, wheat (*Triticum aestivum*), tea (*Camellia sinensis*), *Lotus japonicas*, black poplar (*Populus nigra*) and moss (*Physcomitrella petens*) have been isolated [24, 29–30]. In this study, the STOP1 or ART1 homologous genes had not been found in hydrangea. Moreover, the majority of AtSTOP1/OsART1-regulated genes homologs were unaffected in hydrangea under Al stress. The results suggested that hydrangea may have distinct Al tolerance mechanisms. Previous reports suggested that a tonoplast and a plasma membrane localized aquaporin (HmVALT1 and HmPALT1, respectively) were involved in Al transport in *Hydrangea macrophylla* [11]. However, in our study, their expression was not up-regulated by Al in roots or leaves. That may be because of different tissues (sepals) used in the former study [11].

Organic acids such as oxalate and citrate have been well documented to involve in Al tolerance and accumulation in some plants [7, 13]. Oxalate and citrate play important roles in Al tolerance and oxalate is able to form a complex with Al, detoxifying Al externally and internally [13, 28]. Glycolate/glyoxylate is the efficient precursor for oxalate biosynthesis and is produced during photorespiration or glyoxylate cycle reactions [31]. Except for CS and MDH, other genes involved in the TCA cycle were not changed by Al. These results suggested that citrate biosynthesis was not critical for Al tolerance. Previous studies also documented that it is the transporters rather than biosynthesis of organic acids which are more important for Al tolerance [28].

Al tolerance involves internal mechanisms that allow plants to detoxify Al^3+^ which enters root cells by forming nontoxic organic acid (OA)-Al complexes in the cytosol and/or by sequestering the Al into vacuoles [8]. To facilitate Al hyperaccumulation into the vacuoles, the plasma membrane-localized Al transporter should be present simultaneously. In rice, Nrat1, a member of Nramp family, has been reported to be an Al transporter localized at the plasma membrane [32]. And coordinated plasma membrane/tonoplast Al transport systems are the major contributor to rice’s superior level of Al tolerance compared with other cereal crops [33]. In this study, more than 50 membrane proteins have been up-regulated in the roots. Furthermore, an Nramp aluminum transporter 1 (c198291.graph_c0) was found to be up-regulated in both roots and leaves under Al stress (Tables 5 and 6). In rice, the Nramp aluminum transporter (NRAT1) was involved in Al tolerance by reducing the level of toxic Al in the root cell wall and transporting Al into the root cell, sequestering in the vacuole. The results in our study suggested that the internal Al tolerance mechanism may be also involved in the Al detoxification of hydrangea.

ABC and MATE transporters are also involved in the detoxification of organic compounds. Members of the ABC subfamily are responsible for vacuolar delivery of glutathionated and glucuronated compounds and deposit some large anions into the vacuole [34]. In rice, two ABC transporters, STAR1 and 2 were reported to transport UDP-glucose, which is used for modification of the cell wall [35]. Moreover, Al-induced citrate secretion from roots was mainly mediated by a member of MATE family and was involved in Al tolerance [23, 25, 36]. In this study, we also found that many ABC family transporters and the MATE transporters were up-regulated in roots and leaves under Al stress (Tables 5 and 6).

It is notable that a number of genes relative to energy metabolism, cell wall organization, membrane, DNA damage and metal homeostasis were up-regulated in roots induced by Al in this study. Transcriptomic analysis of *A*. *halleri* and *N*. *caerulescens* with non-accumulator species revealed that a gene set encoding metal-transporters and metal-chelator biosynthestic proteins were highly and constitutively expressed in the hyperaccumulators. In the hyperaccumulator plants, many proteins work simultaneously, such as P-type ATPase (Heavy Metal ATPase 4, HMA4) functions in xylem loading/ unloading and metal tolerance protein 1 (MTP1) functions in vacuolar storage [37]. Four metal tolerance proteins were identified to be up-regulated in roots under Al stress (S9 Table).

Plasma membrane ATPase is the most abundant proteins on the plasma membrane and is reported to be involved in citrate exudation under Al^3+^ toxicity conditions [38]. The process might be achieved through a citrate-proton co-transport system [39]. And most compounds in vacuoles are imported or exported using the electrochemical gradient generated by V-type ATPase [40]. The interesting thing is that many V-type protons ATPase were up-regulated in the roots under Al stress in the study (S10 Table). The results suggested that citrate exudation might be important for Al tolerance in hydrangea.

The cell wall is a major target for Al accumulation and Al^3+^ toxicity in higher plants. The root tip is the primary site of Al^3+^ toxicity, and most of the root Al resides in the cell wall [5, 41]. Al accumulation can increase the rigidity of the cell wall by cross-linking pectin residues which inhibits cell wall loosening needed for root growth [41]. In addition, another protein, expansin which loosen the cell wall during the process of cellular expansion and growth, is very sensitive to Al [42]. In this study, the majority of cell wall proteins were up-regulated and an expansin gene (c172142.graph_c0) was down-regulated in roots under Al stress (S4 Table and S10 Table). The results indicated that increasing the rigidity of cell wall played important roles for Al tolerance in hydrangea. Further functional analysis will reveal their roles of these homologous genes in Al detoxification and accumulation in hydrangea.

## Conclusion

Through RNA-seq data analysis, we constructed about 400,000 high-quality transcripts, which provided information for further investigation of the molecular mechanism of Al accumulation and tolerance in hydrangea and for SNP and SSR markers identifying. The markers will be a rich resource for constructing genetic linkage maps and breeding research in the future. In this study, the results revealed that the roots and leaves may have common and distinct mechanisms of Al responsiveness. Citrate synthase, a key enzyme in the tricarboxylic acid (TCA) cycle, was up-regulated in hydrangea roots and many transporters, including MATE and ABC families, were involved in the process, suggesting that citrate secretion may have its role in the Al tolerance in hydrangea. A plasma membrane Al uptake transporter, Nramp aluminum transporter was up-regulated in roots and leaves under Al stress, and may play an important role in hydrangea Al tolerance by reducing the level of toxic Al. Although the exact roles of these candidate genes remain to be examined, the results in the study provide a platform for further functional analysis of these genes.

## Supporting Information

S1 Table Primers used in the study for quantitative RT-PCR. (XLSX)

S2 Table All unigenes with expression levels (RPKM) in in roots and leaves under Al stress in hydrangea. (XLSX)

S3 Table Annotations of all unigenes that assembled in hydrangea in the study. (XLSX)

S4 Table Up-regulated and Down-regulated genes in roots and leaves under Al stress in hydrangea. Summary of the up-regulated and down-regulated genes in roots and leaves under Al stress.
(XLSX)

S5 Table GO analysis and enrichment of roots under Al stress in hydrangea. (XLSX)

S6 Table GO analysis and enrichment of leaves under Al stress in hydrangea. (XLSX)

S7 Table KEGG analysis and enrichment of roots under Al stress in hydrangea. (XLSX)

S8 Table KEGG analysis and enrichment of leaves under Al stress in hydrangea. (XLSX)

S9 Table The differential expression transporters in roots and leaves in hydrangea. (XLSX)

S10 Table Functional classification of the up-regulated genes in roots in hydrangea. (XLSX)

S11 Table Characterization and distribution of SSRs identified in hydrangea. (XLSX)

S12 Table All the SNPs detected in the study. (XLSX)
